# Insulin Resistance is a Risk Factor for Early Miscarriage and Macrosomia in Patients With Polycystic Ovary Syndrome From the First Embryo Transfer Cycle: A Retrospective Cohort Study

**DOI:** 10.3389/fendo.2022.853473

**Published:** 2022-04-14

**Authors:** Yuanhui Chen, Jiayu Guo, Qingwen Zhang, Cuilian Zhang

**Affiliations:** ^1^ Reproductive Medical Center, People’s Hospital of Zhengzhou University, Zhengzhou, China; ^2^ Reproductive Medical Center, Henan Provincial People’s Hospital, Zhengzhou, China

**Keywords:** insulin resistance, polycystic ovary syndrome, early miscarriage, macrosomia, *in vitro* fertilization

## Abstract

**Objective:**

The objective of the study was to explore the effect of insulin resistance on pregnancy outcomes in patients with polycystic ovary syndrome (PCOS) from the first embryo transfer cycle.

**Design:**

This was a single-center, retrospective, observational cohort study.

**Patients:**

Included in the study were women with PCOS for the first embryo transfer.

**Main Outcome Measures:**

Early miscarriage rate and macrosomia rate were the main outcome measures.

**Results:**

With increased HOMA-IR, the early miscarriage rate (7.14, 13.21, and 16.22%, respectively; *P* = 0.039), macrosomia rate (5.78, 11.79, and 17.58%, respectively; *P* = 0.026) and the incidence of gestational diabetes (GDM) (10.00, 14.50, and 25.67% respectively; *P* = 0.002) significantly increased, while the live birth rate markedly decreased (63.03, 55.27, and 47.88%, respectively; *P* = 0.004). No significant difference was found in clinical pregnancy rate, late miscarriage rate, low birthweight rate and baby gender ratio (all *P >*0.05). After adjusting for confounding factors, HOMA-IR was an independent risk factor of early miscarriage rate and macrosomia rate.

**Conclusion:**

Insulin resistance is an independent risk factor for early miscarriage and macrosomia in PCOS patients during the first embryo transfer cycle. It is essential to give more attention before and after pregnancy for PCOS women with high HOMA-IR.

## Introduction

Polycystic ovary syndrome (PCOS) is a common endocrine disorder that affects about 5–10% reproductive women (1, 2). Common clinical features for PCOS patients include ovulatory disturbances, obesity, hyperandrogenism, hyperinsulinemia, and insulin resistance (IR). IR plays an important role in regulating energy metabolism and follicular growth and development, thus is considered as an important factor in the pathogenesis of PCOS. The incidence of IR in the PCOS population varies from 50 to 70% by different races and regions (3, 4). IR is defined as reduced insulin sensitivity and an increased amount of insulin is needed to perform its normal function. It is generally believed that IR is closely related to obesity. Women with PCOS combined with IR are more prone to metabolic syndrome and cardiovascular disease.

At present, the hyperinsulinemic–euglycemic clamp technique is considered as the gold standard for assessing insulin sensitivity (5), but the complexity and high expense of the method limit its large-scale clinical application. Clinically, the homeostasis model assessment of insulin resistance (HOMA-IR) provides an efficient formula for evaluating B-cell function and insulin sensitivity. It is now a widely used method for assessing IR in many studies (6, 7). For women undergoing assisted reproduction technology, IR is easy to be ignored when fasting blood glucose is normal. A deep understanding about the influence of IR on PCOS may help to explore the pathophysiology of PCOS (8). Moreover, bringing awareness of IR in the reproductive health is crucial for disease management among PCOS women. It was found that although the clinical pregnancy rate with assisted reproduction technology of PCOS was similar to that of non-PCOS patients, the adverse maternal and fetal complications such as the risk of miscarriage, premature delivery, macrosomia, gestational diabetes (GDM) and hypertension were significantly higher (9). Therefore, this study aims to examine the association between IR and clinical pregnancy outcomes by comparing the outcomes of PCOS women with different insulin resistance levels, and discussing the influence of IR on clinical outcomes after the first embryo transfer treatment.

## Materials and Methods

### Study Design and Population

This was a single-center retrospective cohort study approved by the Ethics Committee of the People’s Hospital of Zhengzhou University. Enrolled as subjects of the study were PCOS patients who underwent *in vitro* fertilization (IVF) or intracytoplasmic sperm microinjection (ICSI) procedures for the first time between January 2017 and June 2020 at the Reproductive Medicine Center of People’s Hospital of Zhengzhou University. The diagnosis of PCOS was based on the Rotterdam criteria established in the 2003 Rotterdam consensus workshop, which required that at least two of the following three criteria were met: oligomenorrhea and/or anovulation, clinical and/or biochemical signs of hyperandrogenism, and polycystic ovaries on ultrasound scanning (10).

**Figure 1:**
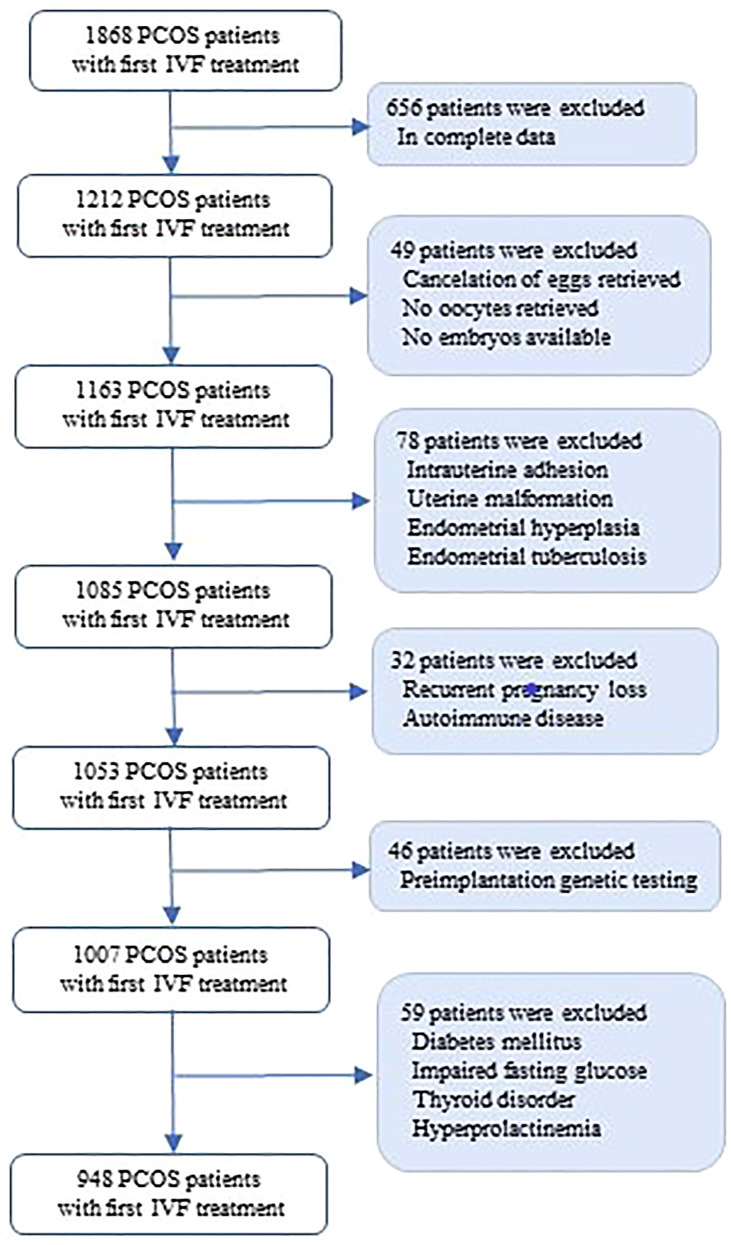
Flow chart of patients’ selection and exclusions.

The exclusion criteria included: 1): cycles with incomplete data; 2) no embryo transfer cycles; 3) with endometrium factors such as intrauterine adhesion and uterine malformation; 4) with recurrent spontaneous abortion and autoimmune disease; 5) with chromosome abnormalities screened by preimplantation genetic screening of preimplantation genetic diagnosis; and 6) other endocrine disorders such as thyroid diseases, diabetes mellitus, impaired fasting glucose and hyperprolactinemia (**Figure 1**). All couples in the study had been given informed consent and signed informed consent for assisted reproduction therapy. This study complied with the basic principles of the Declaration of Helsinki.

Fasting blood glucose and fasting insulin were included in the routine examination of IVF treatment in our center. The two tests were performed six months before the start of ovarian stimulation, and in the same laboratory. As there is no consensus about the cutoff value of IR at present, the patients was divided into three groups according to 25th and 75th quartile of the HOAM-IR in this study: Group 1: HOMA-IR ≤1.87 (n = 238); group 2: 1.87 <HOMA-IR <4.28 (n = 474); and group 3: HOMA-IR ≥4.28 (n = 236). The insulin resistance index was calculated using the HOMA-IR according to the following formula: HOMA-IR = fasting blood glucose × fasting insulin/22.5. The unit of fasting blood glucose was mmol/L and the unit of fasting insulin was μ U/ml.

### Ovarian Stimulation Protocols

In this study, the controlled ovulation induction protocol was conducted by the same team according to the condition of the patients. All the women underwent either GnRH agonist or flexible GnRH antagonist protocol.

### GnRH Agonist Protocol

For the GnRH agonist protocol, 30 to 35 days after a single injection of 3.75 mg of long-acting GnRH agonist (Diphereline, Ipsen, Tianjin) on the second or third day of menstrual cycle, or injection of the short-acting GnRH agonist (Decapeptyl, 0.1 mg/d, Germany ferring) for 14 to 18 days began in the middle luteal phase of the previous menstrual cycle. Once the condition reached the downregulation standard, a dose of 75–300 IU gonadotropin (Gn) was administered based on the age, ovarian reserve, body mass index (BMI), and anti-Mullerian hormone (AMH) level of the patient. Gonadotropin doses were adjusted according to ovarian response and hormone levels after 4 to 5 days. Urinary human chorionic gonadotropin (hCG) was administered subcutaneously for triggering when at least two follicles measured ≥18 mm or three follicles measured ≥17 mm. A dose of 4,000 to 10,000 IU of hCG (Lizhu Pharmaceutical Trading, China) was given to induce ovulation depending on peak estradiol level and age. Oocyte retrieval guided by vaginal ultrasound was performed 36–37 h later.

### GnRH Antagonist Protocol

Gn was injected from the second or third day of menstruation, and the starting dose of Gn was the same as above. Follicular size and hormone levels were monitored after four or five days of Gn treatment. A daily dose of 0.25 mg GnRH antagonist was initiated when a dominant follicle reached a mean diameter of 12 mm or estrogen level ≥200 ng/L or when blood luteinizing hormone (LH) levels began to show a notable upward trend. The dose was administered until the day of hCG administration. When at least two follicles measured ≥18 mm or three follicles measured ≥17 mm, a dose of 4,000 to 10,000 IU hCG was administered subcutaneously for triggering. Oocyte retrieval guided by vaginal ultrasound was performed 35–36 h later.

### Embryo Transfer and Luteal Support

IVF/ICSI fertilization was performed depending on male semen parameters. On the 3rd to 5th day after oocyte retrieval, 1–2 high-quality cleavage embryos or blastocysts were selected for embryo transfer. During the frozen embryo transfer cycle, the endometrial preparation protocol was selected individually according to the condition of the patient, and 1–2 cleavage embryos or blastocysts should be transferred timely after the endometrial transformation. The hCG level in peripheral blood was measured on the 14th day after embryo transfer. Clinical pregnancy was defined as the presence of at least one intrauterine gestational sac on the 4–5 weeks after transfer. Luteal support drugs were discontinued in non-pregnant patients, and luteal support drugs were continued in pregnant patients until 8–10 weeks of pregnancy.

### Outcomes

The primary outcomes of this study were the early miscarriage rate, macrosomia birth rate, and live birth rate. Live birth was defined as the complete removal or delivery of the fertilized product from the mother after more than 28 weeks of gestation with the presence of respiration or any signs of life (heartbeat, umbilical cord pulsation, voluntary muscle movement) after separation from the mother. Early miscarriage was defined as embryo loss before 12 weeks of pregnancy. Low birth weight was defined as fetal birth weight <2,500 g. Macrosomia was defined as birth weight ≥4,000 g.

### Statistical Analysis

All measurement data were expressed by mean ± standard deviation (mean ± SD). One-way ANOVA was used for comparison between groups. All counting data were expressed by percentage (%), and chi-squared test was used to compare the count data between groups. Logistic regression model was used for multivariate analysis.

All statistical management and analyses were performed using SPSS software, version 24.0. A two-sided *P-*value <0.05 was considered statistically significant.

## Results

### Study Population

A total of 948 PCOS women who underwent first embryo transfer cycle and met the study inclusion and exclusion criteria were enrolled (**Figure 1**). According to the 25th and 75th of HOMA-IR, all the patients were divided into three groups: group 1 with HOMA-IR ≤1.87 (n = 238), group 2 with HOMA-IR between 1.87 and 4.28 (n = 474), and group 3 with HOMA-IR ≥4.28 (n = 236).

### Patient Demographic and Characteristics

**Table 1** showed the demographic and clinical characteristics among the three groups. The mean HOMA-IR was significant different in the three groups (1.43 ± 0.35, 2.92 ± 0.68, 6.72 ± 2.78, respectively; *P <*0.001). The BMI (22.1 ± 2.9, 24.9 ± 3.4, 28.3 ± 3.4, respectively; *P <*0.001) and basal testosterone (T) (0.41 ± 0.2, 0.42 ± 0.19, 0.46 ± 0.22; *P* = 0.020) significantly increased with HOMA-IR. In group 3, obese patients (BMI ≥28 kg/m^2^) accounted for as high as 52.1%. The level of AMH, basal follicular stimulation hormone (FSH) and basal luteinizing hormone (LH) decreased significantly among the three groups. There were no significant differences in age, duration of infertility, type of infertility and fertilization method (*P >*0.05).

**Table 1:** Comparison of demographic and clinical characteristics of the three groups.

Item	Group 1	Group 2	Group 3	*P*
No. of cases	238	474	236	
Age (year)	29.1 ± 3.9	28.9 ± 3.6	28.5 ± 4.2	0.208
HOMA-IR	1.43 ± 0.35	2.92 ± 0.68	6.72 ± 2.78	<0.001
BMI (kg/m^2^)	22.1 ± 2.9	24.9 ± 3.4	28.3 ± 3.4	<0.001
<24	76.5 (182/238)	43.0 (204/474)	9.3 (22/236)	
24–27.9	19.7 (47/238)	38.9 (184/474)	38.6 (91/236)	
≥28	3.8 (9/238)	18.1 (86/474)	52.1 (123/236)	
AMH (ng/ml)	9.2 ± 4.9	8.3 ± 4.5	7.2 ± 4.1	<0.001
FSH (IU/L)	6.1 ± 1.5	5.7 ± 1.3	5.5 ± 1.4	<0.001
LH (IU/L)	10.5 ± 5.8	8.7 ± 4.7	7.6 ± 4.2	<0.001
T (ng/ml)	0.41 ± 0.2	0.42 ± 0.19	0.46 ± 0.22	0.020
Duration of infertility (year)	2.5 ± 0.1	2.5 ± 0.1	2.5 ± 0.2	0.068
Type of infertility (%)				0.103
Primary	63.9 (152/238)	61.8 (293/474)	69.9 (165/236)	
Secondary	36.1 (86/238)	38.2 (181/474)	30.1 (71/236)	
Methods of ART (%)				0.314
IVF	84.5 (201/238)	87.6 (415/474)	89.0 (210/236)	
ICSI	15.5 (37/238)	12.4 (59/474)	11.0 (26/236)	

### Ovarian Stimulation and First Embryo Transfer Results

As shown in **Table 2**, with increased HOMA-IR, the starting dosage of Gn, the total dosage of Gn and the duration of Gn became higher, while the number of oocytes retrieved, number of mature oocytes, number of normal fertilization oocytes, number of available embryos and number of good embryos became significantly lower (*P <*0.05). No statistically significant difference was observed in type of protocol.

**Table 2:** Ovarian stimulation characteristics among the three groups.

Item	Group 1	Group 2	Group 3	*P*
No. of cases	238	474	236	
Protocol (%)				0.078
GnRH agonist protocol	83.2 (198/236)	86.5 (410/474)	90.3 (213/236)	
GnRH antagonist protocol	16.8 (40/236)	13.5 (64/474)	9.7 (13/236)	
Starting dosage of Gn (IU)	122.1 ± 27.7	131.9 ± 29.2	142.9 ± 31.7	<0.001
Total dosage of Gn (IU)	1,741.4 ± 87.6	2,200.8 ± 1,202.6	2,880.0 ± 1,254.2	<0.001
Duration of Gn (d)	11.2 ± 3.2	12.2 ± 3.7	13.7 ± 3.7	<0.001
No. of oocytes retrieved	16.0 ± 7.7	14.8 ± 7.9	13.8 ± 7.8	0.011
No. of mature oocytes	13.6 ± 7.0	12.7 ± 7.1	11.8 ± 6.8	0.017
No. of normal fertilization oocytes	9.6 ± 5.6	9.0 ± 5.5	8.1 ± 5.2	0.009
No. of available embryos	7.7 ± 4.8	7.6 ± 5.0	6.7 ± 4.5	0.048
No. of good embryos	4.1 ± 2.5	3.8 ± 2.4	3.6 ± 2.1	0.036

After the first embryo transfer, the type of transfer (fresh cycle or frozen cycle), number of embryos transferred and the thickness of endometrium were comparable among the groups (*P >*0.05). With increased HOMA-IR, the early miscarriage rate (7.14, 13.21, and 16.22%, respectively; *P* = 0.039), macrosomia rate (5.78, 11.79, and 17.58%, respectively; *P* = 0.026) and the incidence of GDM (10.00, 14.50, and 25.67% respectively; *P* = 0.002) significantly increased, while the live birth rate markedly decreased (63.03, 55.27, and 47.88%, respectively; *P* = 0.004). No macrosomia baby was born in twin pregnancy patients. Furthermore, the live birth rate of single baby was prominently lower, while the rate of twin live birth was comparable. No significant difference was found in clinical pregnancy rate, late miscarriage rate, low birth weight rate, and baby gender ratio (**Table 3**).

**Table 3:** Outcomes of first embryo transfer cycle.

Item	Group 1	Group 2	Group 3	*P*
No. of cases	238	474	236	
Type of transfer (%)				0.128
Fresh cycle	43.3 (103/238)	47.3 (224/474)	52.5 (124/236)	
Frozen cycle	56.6 (135/238)	52.7 (250/474)	47.4 (112/236)	
No of embryo transferred	1.47 ± 0.50	1.47 ± 0.50	1.50 ± 0.50	0.631
Type of transfer embryos (%)				0.021
cleavage	57.1 (136/238)	55.9 (265/474)	66.5 (157/236)	
blastocyst	42.9 (102/238)	44.1 (209/474)	33.5 (79/236)	
Endometrium (mm)	9.8 ± 1.9	10.0 ± 2.0	10.1 ± 2.2	0.337
Clinical pregnancy rate (%)	70.59 (168/238)	67.09 (318/474)	62.71 (148/236)	0.188
Early miscarriage rate (%)	7.14 (12/168)	13.21 (42/318)	16.22 (24/148)	0.039
Late miscarriage rate (%)	2.38 (4/168)	2.83 (9/318)	5.41 (8/148)	0.258
Live birth rate (%)	63.03 (150/238)	55.27 (262/474)	47.88 (113/236)	0.004
Single live birth rate (%)	50.84 (121/238)	44.73 (212/474)	38.56 (91/236)	0.027
Low birth weight rate (%)	5.78 (7/121)	6.60 (14/212)	9.89 (9/91)	0.478
Macrosomia rate (%)	5.78 (7/121)	11.79 (25/212)	17.58 (16/91)	0.026
Twin live birth rate (%)	12.18 (29/238)	10.55 (50/474)	9.32 (22/236)	0.597
Low birthweight rate (%)	31.03 (18/58)	44 (44/100)	50 (22/44)	0.125
Macrosomia rate (%)	0	0	0	
GDM (%)	10.00 (15/150)	14.50 (38/262)	25.67 (29/113)	0.002
Baby gender ratio (male/female)	1.11	1.17	1.11	0.948
Total Male	94	168	71	
Total Female	85	144	64	

Multivariate logistic regression analysis was performed to explore the risk factors of early miscarriage rate and macrosomia rate. The regression model included the following factors: age, HOMA-IR, BMI, AMH, number of available embryos, number of embryos transferred, type of transfer embryo and endometrial thickness. The results showed that HOMA-IR was an independent risk factor of early miscarriage rate and macrosomia rate. Compared with group 1, the group 2 and group 3 had significantly higher early miscarriage rate (group 2, a*O*R = 1.640, 95% CI: 1.101–2.443, *P* = 0.015; group 3, a*O*R = 1.685, 95% CI: 1.049, 2.708, *P* = 0.031) and macrosomia rate (group 2, a*O*R = 1.983, 95% CI: 1.089–3.611, *P* = 0.025; group 3, a*O*R = 2.218, 95% CI: 1.149–4.281, *P* = 0.018). The details are shown in **Table 4**.

**Table 4:** Logistic regression analysis to account for confounding variables of early miscarriage and macrosomia.

	Early miscarriage rate			Macrosomia rate		
	B	*aOR* (95% confidence interval)	*P*	B	*aOR* (95% confidence interval)	*P*
HOMA-IR			0.039			0.035
Group 1		Ref (1)			Ref (1)	
Group 2	0.495	1.640 (1.101, 2.443)	0.015	0.685	1.983 (1.089, 3.611)	0.025
Group 3	0.522	1.685 (1.049, 2.708)	0.031	0.779	2.218 (1.149, 4.281)	0.018

## Discussion

In the present study, we found that HOMA-IR was associated with early miscarriage, macrosomia, live birth rate, and the incidence of GDM. With increasing of HOMA-IR, the early miscarriage rate, the macrosomia rate and the prevalence of GDM elevated remarkedly, and the live birth rate decreased significantly in their first embryo transfer. The influences still remained after adjusting for the following factors: age, BMI, AMH, number of available embryos, number of embryos transferred, type of transfer embryo and endometrial thickness.

Further, we found that with increased HOMA-IR, there were significant decreasing in number of oocytes retrieved, number of available embryos, and number of good embryos. We suspect that with fewer available embryos and good embryos, reduced chance for embryo selection in the first embryo transfer cycle might lead to adverse pregnancy outcomes, and high HOMA-IR may be detrimental to the oocyte and embryo quality.

### IR and Early Miscarriage

Several previous studies have shown that PCOS patients had a higher miscarriage rate than non-PCOS patients in IVF treatment. Su et al. found that women with PCOS had an increased risk miscarriage (aOR 1.629, 95% CI 1.240–2.141) for the first IVF treatment (11). A meta-analysis including twenty-nine studies also demonstrated that PCOS women had higher risks of miscarriage (OR 1.41, 95% CI 1.04–1.91) than control group (9). Due to the complexity of endocrine disorders in PCOS population, no clear indicators exist concerning the exact risk factors for adverse pregnancy outcomes. A meta-analysis found that high BMI (OR 1.48, 95% CI [1.32, 1.67], MD = 1.35, 95% CI [0.58,2.12]) and insulin resistance (MD = 0.32, 95% CI [0.15, 0.49]) were associated with an increased risk of miscarriage in PCOS patients undergoing ART (12). Li et al. found that IR was an independent risk factor for spontaneous abortion (13), which was consistent with our study. However, the definition of IR used in our study was different compared with previous studies. In the study of Li et al. (13), patients with HOMA-IR greater than 4.5 were classified as IR, while in our study HOMA-IR was grouped by the percentile. At present time, there is no consensus on the definition of IR, as previous studies have variously defined IR with the level of HOMA-IR. In this study, we grouped the patients by 25th quantile and 75th quantile of HOMA-IR and explore the relationship between HOMA-IR and adverse pregnancy outcomes.

IR might affect early miscarriage through downstream physiological changes. IR or hyperinsulinemia may affect the secretion of androgen, and excess androgen can aggravate endocrine disorders and follicular dysplasia, which may further result in poor quality eggs and embryos. Besides, from an *in vivo* study, hyperandrogenism and insulin resistance could induce mitochondria-mediated damage and result in an imbalance between oxidative and antioxidative stress responses in the gravid uterus, which correlates with high abortion risk (14). An experiment in pregnant rats suggested that deleterious effects of hyperandrogenism and insulin resistance on fetal survival were related to placental mitochondrial abnormalities and elevated reactive oxygen species production (15). Additionally, gut microbiota dysbiosis can promote metabolism, immune response through interaction with the external environment, which may closely relate with IR in PCOS patients and cause adverse pregnancy outcomes (16). Other factors, such as serum testosterone and serum chemerin level, might also contribute to the early abortion in PCOS women (17, 18).

### IR Affecting Macrosomia

In this study, we found that macrosomia rate and the incidence of GDM significantly increased with HOMA-IR elevation, and the influence was still remained after adjusting for the possible confounding factors. A meta-analysis including fifty-nine studies of Chinese PCOS women suggested that the estimates of GDM and macrosomia among women with PCOS were significantly higher than those in women without PCOS (all *P <*0.05). Further subgroup analysis found that PCOS women with pre-pregnancy insulin resistance were at an increased risk for GDM and macrosomia (all *P <*0.05) (19). A retrospective cohort study including 1,357 pregnant women with PCOS and 6,940 without PCOS suggested that PCOS women had a higher rate of macrosomia (9.14% vs 6.64%, *P* = 0.008), and the difference was prominent among obese PCOS women with no significant difference (18.92% vs 8.00%, *P* = 0.15) (20).

At present, a large number of studies have found that maternal weight was a high-risk factor of macrosomia (21–24). Additionally, a study found that insulin resistance was a link between maternal overweight and fetal macrosomia in nondiabetic pregnancies (25). Study has shown that there was a significant positive correlation between maternal weight and HOMA-IR (r = 0.248, *P <*0.05) (26). In our study, the BMI increased significantly in accordance with HOMA-IR (*P <*0.001), and more than half women (52.1%) were obese (BMI ≥28) when HOMA-IR was more than 4.28. PCOS is commonly characterized by endocrine disorder such as insulin resistance, hyperandrogenism, and obesity. Obesity and insulin resistance are closely interrelated.

Macrosomia has short-term and long-term adverse health effects and is thus an important public health concern. A murine model suggested that neonatal macrosomia was an independent risk factor of adult metabolic syndrome (27). Another research including 1,767 infants explored the risk of childhood under 3 years, and found that obesity for macrosomic babies was 3.74 (1.96–7.14) and 1.64 (0.89–3.00) times higher based on weight-for-age and BMI-for-age, respectively (28). It is essential to explore the risk factors and possible mechanisms of macrosomia. The higher rate of macrosomia maybe associated with the greater risk of GDM in PCOS patients. PCOS patients had a high incidence of GDM and prevalence of GDM diagnosis in the first trimester, especially in patients with obesity and insulin resistance (29–31). In our study, the incidence of GDM significantly increased with HOMA-IR, which was in accordance with the occurrence of macrosomia. However, even with no GDM during pregnancy, there still was an increased risk of macrosomia with insulin resistance (aOR:1.71; 95% CI: 1.12–1.97) (32). In addition, during pregnancy, maternal tissues become increasingly insensitive to insulin in order to liberate nutritional supply to the growing fetus. Thus, IR might be an important risk factor for macrosomia among PCOS patients.

### Strengths and Limitations

To our best knowledge, this is the first study to explore the effects of insulin resistance both on early miscarriage and macrosomia in PCOS patients during their first embryo transfer cycles. Most of the previous studies have compared the influence of IR on PCOS patients and non-PCOS patients. It provides valuable data support for clinical consultation and new ideas for future clinical and basic research. This study also has certain limitations that should be noticed. First, this study was designed as a retrospective cohort study, and thus limited its scope to explore the relevant biological mechanism by which insulin resistance affects pregnancy outcomes. Additionally, the assessment of HOMA-IR has some limitations (33). HOMA-IR reflects predominantly hepatic insulin resistance, while peripheral insulin resistance is better described by oral glucose tolerance test-derived (OGTT) insulin resistance indices. However, the data of OGTT and other maternal complications during pregnancy were not studied in this study since the data were collected retrospectively.

## Conclusion

In summary, this study showed that insulin resistance was an independent risk factor for early miscarriage and macrosomia in PCOS patients during the first embryo transfer cycle. The early miscarriage rate and macrosomia rate were significantly higher with the increasing of HOMA-IR. Therefore, for PCOS patients with high insulin level, it is essential to give effective treatment before pregnancy, and the perinatal period may require more attention from obstetricians and pediatricians.

## Data Availability Statement

The raw data supporting the conclusions of this article will be made available by the authors, without undue reservation.

## Ethics Statement

The studies involving human participants were reviewed and approved by the Zhengzhou University and the Henan Provincial People’s Hospital. Written informed consent for participation was not required for this study in accordance with the national legislation and the institutional requirements.

## Author Contributions

YC designed the study. JG and QZ were involved in the data extraction and analysis. CZ was responsible for providing data and guiding research. All authors listed have made a substantial, direct, and intellectual contribution to the work and approved it for publication.

## Funding

This work was supported by the National Natural Science Foundation of China (NSFC) (project number: U2004130).

## Conflict of Interest

The authors declare that the research was conducted in the absence of any commercial or financial relationships that could be construed as a potential conflict of interest.

## Publisher’s Note

All claims expressed in this article are solely those of the authors and do not necessarily represent those of their affiliated organizations, or those of the publisher, the editors and the reviewers. Any product that may be evaluated in this article, or claim that may be made by its manufacturer, is not guaranteed or endorsed by the publisher.
